# Clofazimine enteropathy: a case of pigmentation of the whole small intestine caused by clofazimine

**DOI:** 10.1055/a-2344-8244

**Published:** 2024-07-03

**Authors:** Huan Zhang, Qinghui Peng, Xinhua Zhao, Yuanjing He, Jun Liu

**Affiliations:** 1 536557 Department of Gastroenterology, Mianyang Central Hospital, Mianyang, China

Clofazimine is mainly used for the treatment of neoplastic leprosy, and can also be used in combination with other anti-tuberculosis drugs to treat multidrug-resistant tuberculosis.

As is well known, clofazimine can within weeks cause skin pigmentation in 75% to 100% of patients, or, uncommonly, ichthyosis 11 . One of the most serious gastrointestinal side effects of clofazimine is deposition of black-purple crystals in the small bowel lamina propria, which leads to hyperpigmentation in the intestines. This most serious side effect of clofazimine, although rare, can result in severe or even fatal enteropathy 22 . We report a case of intestinal injury caused by clofazimine. After timely diagnosis and cessation of medication, the patient’s condition has significantly improved.

Our 32-year-old patient with drug-resistant tuberculosis developed abdominal pain after receiving anti-tuberculosis treatment with pyrazinamide, clofazimine, levofloxacin, and iminicotinamide for 16 months. Physical examination revealed that the skin on his back was reddish brown ( Fig. 1Fig. 1 ), and the anterior tibial skin of both his lower limbs showed fish-scale-like changes ( Fig. 2Fig. 2 ). Colonoscopy showed melanosis in the terminal ileum. The pathological report stated that tissue cells could be seen in the lesion area, and crystal-like substances could be seen inside ( Fig. 3Fig. 3 ). Capsule endoscopy showed continuous pigmentation of the jejunum and ileum, with pigmentation of the jejunum being prominent ( Video 1Video 1 ).

**Fig. 1:**
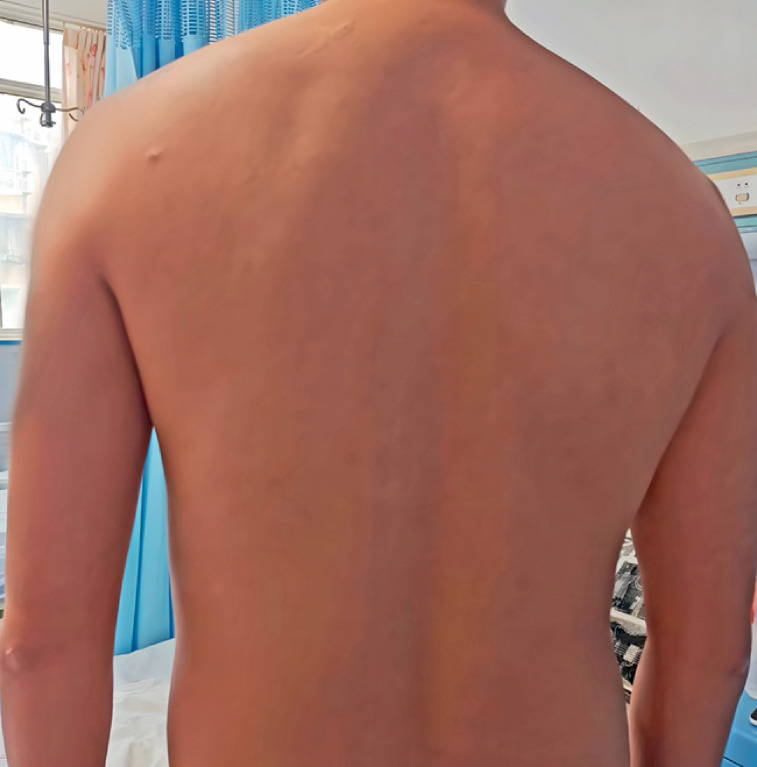
**Fig. 1** Clofazimine caused reddish-brown pigmentation of the skin on the back of a patient with intestinal injury.

**Fig. 2:**
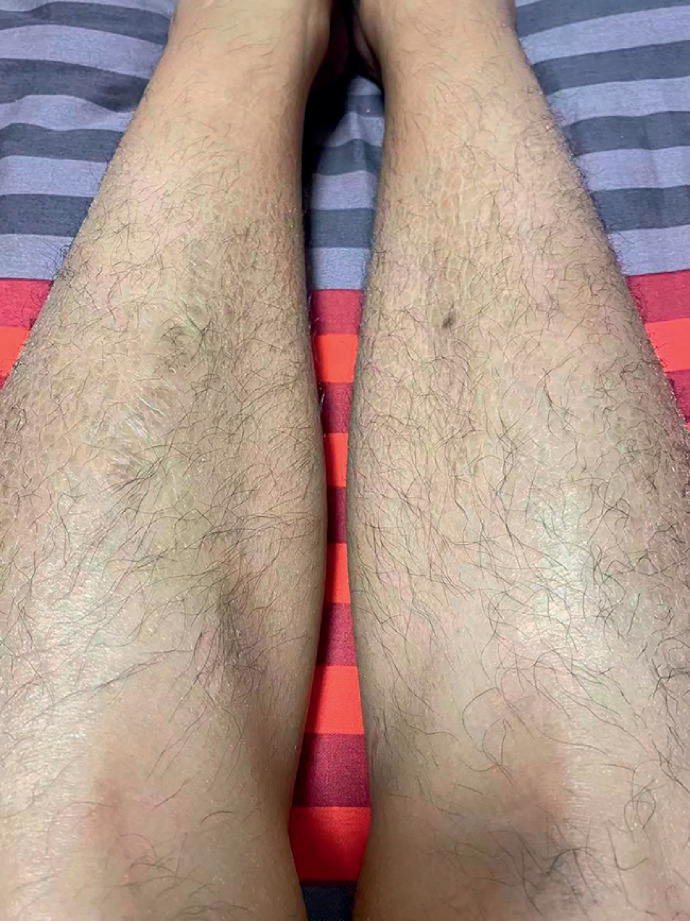
**Fig. 2** Clofazimine caused fish-scale-like changes (ichthyosis) in the skin of both lower limbs in a patient with intestinal injury.

**Fig. 3:**
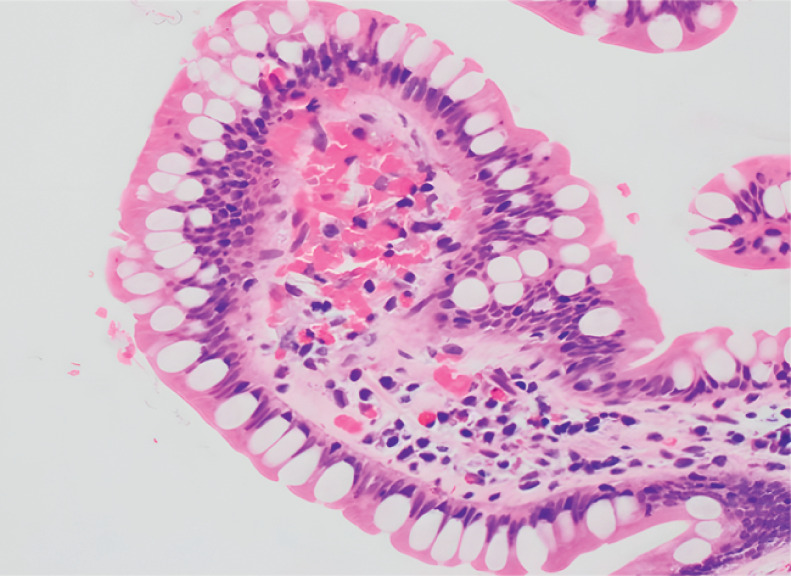
**Fig. 3** The pathological report on the biopsy of the distal segment of the ileum identified moderate chronic inflammation of the mucosa, with infiltration of lymphocytes, plasma cells, and scattered eosinophils. Tissue cells can be seen in the lesion area, and crystal-like substances can be seen inside.

Continuous pigmentation of the jejunum and ileum, with pigmentation of the jejunum being prominent.Video 1Video 1

Clofazimine treatment was stopped, and within 5 months the patient’s abdominal pain disappeared and the pigmentation and fish-scale-like changes in the back and lower limbs decreased.

Endoscopy_UCTN_Code_CCL_1AB_2AH_3AB

## Notes

Endoscopy E-Videos https://eref.thieme.de/e-videos
*E-Videos* is an open access online section of the journal *Endoscopy* , reporting on interesting cases and new techniques in gastroenterological endoscopy. All papers include a high-quality video and are published with a Creative Commons CC-BY license. Endoscopy E-Videos qualify for HINARI discounts and waivers and eligibility is automatically checked during the submission process. We grant 100% waivers to articles whose corresponding authors are based in Group A countries and 50% waivers to those who are based in Group B countries as classified by Research4Life (see: https://www.research4life.org/access/eligibility/ ). This section has its own submission website at https://mc.manuscriptcentral.com/e-videos .
